# Knowledge-practice decoupling in residential chemical indoor-air pollution: a cross-sectional survey in Lebanon

**DOI:** 10.3389/fpubh.2026.1971900

**Published:** 2026-10-14

**Authors:** Wissam Ghach, Rina Salhab, Jana K Alwattar, Sara Abou Ibrahim, Nisreen Alwan, Emran Alotaibi

**Affiliations:** 1 Department of Public Health, Canadian University Dubai, Dubai, United Arab Emirates; 2 Department of Pharmaceutical Sciences, School of Pharmacy, Lebanese International University, Beirut, Lebanon; 3 Faculty of Arts and Sciences, Modern University for Business and Science, Damour, Lebanon; 4 Department of Environmental and Public Health, Abu Dhabi University, Abu Dhabi, United Arab Emirates

**Keywords:** chemical indoor pollution, household practices, knowledge, Lebanon, preventive attitudes, residential indoor air, symptom recognition

## Abstract

**Introduction:**

Residential chemical indoor-air pollution arises from combustion, smoking residues, fragranced products, cleaning chemistry, pesticides, building materials and ventilation behavior, yet household-level evidence from Lebanon remains limited. This study assessed knowledge, preventive attitudes, household practices and symptom recognition, with particular attention to whether knowledge translated into exposure-relevant behavior.

**Methods:**

This secondary cross-sectional analysis used a non-probability convenience sample of 495 completed self-administered questionnaires collected between 8 November 2025 and 10 April 2026. Evidence-aligned scoring was used for knowledge and preventive attitudes, while practice items were separated into mitigation and source-frequency indices. Descriptive statistics, internal-consistency estimates, Wilson 95% confidence intervals, Spearman correlations, robust linear models and robust logistic models were used.

**Results:**

Respondents were predominantly young, female and university educated. Mean knowledge and preventive-attitude scores were 47.3 and 48.8 on 0-100 scales; the mean mitigation index was 66.3 and the mean source-frequency index was 58.4. Knowledge was moderately associated with preventive attitudes but only weakly associated with mitigation behavior. Higher preventive-attitude scores were associated with lower source-frequency, lower odds of regular indoor smoking and a higher mitigation-minus-source difference score. Symptom recognition was stronger for respiratory and neurological than for ocular, irritant and dermatological manifestations.

**Discussion:**

The findings indicate knowledge-practice decoupling within this sample: mitigation behaviors coexisted with frequent source-related activities, and knowledge alone did not explain household practice. Because the study used a non-probability, self-reported cross-sectional sample, results are sample-specific and do not establish national prevalence or causal effects. Source-specific risk communication and representative studies incorporating objective exposure measures are warranted.

## 1 Introduction

Residential indoor air has become a central exposure environment for environmental health because people spend large fractions of daily life inside buildings and a substantial share of that time at home. The home is not a passive shelter from outdoor pollution: it is also a chemically active microenvironment where combustion, tobacco smoke, fragrance chemistry, cleaning products, pesticides, moisture, furnishing materials and ventilation patterns jointly determine exposure. Time-activity evidence from the National Human Activity Pattern Survey established the importance of indoor microenvironments for population exposure assessment (1). Subsequent residential measurement syntheses identified multiple chemical contaminants of health relevance in homes, including fine particles, nitrogen dioxide, carbon monoxide, formaldehyde, acrolein, chloroform and other volatile organic compounds (VOCs) (2, 3, 31). This evidence base reframes household air pollution as a continuous mixture problem rather than a problem confined to obvious smoke or odor episodes.

The chemical complexity of homes is partly driven by everyday activities that are viewed as normal, comfortable or hygienic. Gas cooking can emit nitrogen oxides, carbon monoxide, formaldehyde and benzene; recent residential studies show that gas and propane stoves can elevate nitrogen dioxide throughout dwellings and that combustion from stoves can generate detectable benzene, including in rooms away from kitchens (4–6). Epidemiological synthesis has linked gas cooking and indoor nitrogen dioxide with childhood wheeze and asthma risk (7). Household cleaning and air-freshening practices add another pathway. Cleaning products and air fresheners can emit primary VOCs and can also participate in ozone-terpene reactions that generate secondary pollutants such as formaldehyde and Secondary organic aerosol (8–10). Contemporary reviews emphasize that cleaning chemistry can improve hygiene while simultaneously creating exposure risks when product selection, mixing, application and ventilation are poorly controlled (11). Fragranced consumer products and air fresheners are particularly important because their use is often motivated by perceived freshness, despite evidence that they emit mixtures of VOCs and that discontinuation can measurably reduce marker compounds such as d-limonene (12–14).

Tobacco smoke adds direct and residual exposure pathways. Second-hand smoke is an established indoor pollutant mixture, but third-hand smoke is less visible and more easily underestimated. Nicotine and other constituents deposited on indoor surfaces can persist, desorb, react with indoor oxidants and produce tobacco-specific nitrosamines, creating exposure through inhalation, ingestion and dermal contact long after active smoking stops (15–17). This residue-based mechanism is important for household risk communication because the absence of visible smoke does not indicate the absence of exposure. Formaldehyde represents another invisible risk domain. It arises from pressed-wood products, furnishings, combustion and indoor chemistry, and its indoor relevance has been extensively reviewed in toxicological and exposure literature (18, 19). These pollutant classes share a public-health communication challenge: they are generated through ordinary domestic routines, are not always perceptible by smell or sight, and are affected by source control and ventilation decisions that vary by climate, housing and cultural practices.

Despite extensive exposure science, the behavioral interface remains comparatively underdeveloped. Measurement studies identify pollutants and sources; prevention requires understanding how residents interpret those sources, which actions are considered protective, and where knowledge fails to translate into practice. Knowledge-attitude-practice (KAP) and knowledge-attitude-behavior frameworks are useful because they examine the pathway from risk awareness to intention and action, while allowing identification of barriers and misconceptions. More explicit stage-based frameworks, including the Protective Action Decision Model and the Theory of Planned Behavior, distinguish risk appraisal, social and control beliefs, behavioral intention and implementation. An integrated Protective Action Decision Model and the Theory of Planned Behavior (PADM-TPB) analysis of PM2.5 preventive-measure adoption in urban Thailand showed that protective action can remain incomplete even when risk awareness is present because intention and action depend on additional appraisal and control pathways (20). Existing community studies likewise suggest that knowledge alone is insufficient. In Singapore, a national cross-sectional study on indoor air quality showed that public knowledge, attitudes and behaviors differ across population groups and that behavior change requires more than general awareness (21). In Italy, a household chemical indoor-pollution KAP survey reported that attitudes and behavior were not a simple function of knowledge and called for targeted educational programs (22). In Kuwait, knowledge and attitudes toward indoor pollution were assessed among university students and teachers, with findings supporting the need for institutional education on indoor sources (23). More recent behavioral work using integrated knowledge-attitude-behavior models indicates that protective actions are shaped by attitudes, perceived barriers and perceived benefits, not only factual knowledge (24).

The regional evidence gap is particularly relevant for Lebanon and the wider Eastern Mediterranean. Air-pollution KAP research in Lebanon has largely focused on professional groups such as physicians, where studies have highlighted training and counseling needs around air-pollution health effects (25); however, professional awareness does not substitute for household-level evidence on how residents interpret indoor chemical sources, especially in settings where gas cooking, incense, candles, air fresheners, indoor smoking, intermittent ventilation, generator-related infiltration and diverse household products may coexist. Peer-reviewed household evidence on residential chemical indoor-air pollution in Lebanon remains scarce, and regional studies have rarely integrated knowledge, attitudes, protective practices, source-related behaviors and symptom recognition in a single analytic framework. This gap limits the design of locally credible intervention messages because generic advice such as improving ventilation or maintaining cleanliness can conflict with air-conditioning practices, energy constraints, thermal comfort, product preferences and family smoking norms.

The present study addresses this gap through a secondary cross-sectional analysis of 495 completed questionnaires from respondents in Lebanon. The analysis had four objectives: to characterize the sample and describe evidence-aligned knowledge and preventive attitudes; to separate mitigation practices from source-frequency practices rather than treating household behavior as a single unidirectional construct; to identify item-level uncertainty around sources and mechanisms such as third-hand smoke, formaldehyde, gas combustion and indoor-outdoor exposure assumptions; and to examine associations among knowledge, attitudes, household practices and symptom-recognition literacy. The central hypothesis was that general knowledge would be associated with preventive attitudes but would not fully explain household practices, whereas source-specific attitudes and smoking-related factors would show stronger associations with exposure-relevant behavior. Because the sample was non-probability and demographically concentrated, all descriptive estimates are interpreted as characteristics of the analytic sample rather than population prevalence estimates for Lebanon.

## 2 Materials and methods

### 2.1 Study design and reporting approach

This study used a secondary cross-sectional design to analyze a structured self-administered questionnaire on residential chemical indoor-air pollution in Lebanon. Because data were collected at a single point in time from each respondent, the findings describe patterns and associations and should not be interpreted as temporal or causal effects. The reporting and analytical structure followed the general principles of the Strengthening the Reporting of Observational Studies in Epidemiology (STROBE) statement for cross-sectional studies, including description of the study population, variables, data-cleaning procedures, statistical methods, potential sources of bias and limits to generalizability (26).

The analytic dataset contained 495 completed responses and 51 variables recorded between 8 November 2025 and 10 April 2026. Each row represented one respondent. The available secondary-analysis documentation did not contain a probability-based sampling frame, sampling weights or the recruitment denominator required to calculate a response rate. The dataset was therefore treated as a non-probability convenience sample, and all percentages and model estimates describe the respondents in this sample rather than the broader Lebanese population.

The analysis was structured to support transparent reporting of the study population, questionnaire domains, data-cleaning procedures, scoring rules, measurement properties, statistical models and sensitivity analyses. No pollutant concentrations or clinical outcomes were measured.

### 2.2 Data source, participants and setting

The study included respondents with completed questionnaire records from the general community in Lebanon. The questionnaire collected information on demographic characteristics, knowledge of indoor chemical pollution, preventive attitudes, household practices and recognition of symptoms that may be associated with chemical exposure. Records were retained when the information needed to construct the principal analytical domains was available.

Demographic variables included gender, age, governorate of residence, educational level, current smoking status, presence of children in the household and previous hospitalization because of chemical poisoning. These variables were used for descriptive analyses and, where specified, as covariates in multivariable models.

Names, email addresses and other direct identifiers were not used in the statistical analyses and were excluded from the analytic dataset. No record was excluded solely because identifying fields were present in the original survey file.

For regression analysis, governorate was grouped into Beirut, Mount Lebanon and other governorates to reduce instability from small cells in individual regional categories. This grouping was analytical only and was not intended to imply geographic representativeness.

### 2.3 Data cleaning and standardization

All questionnaire responses were reviewed and standardized before analysis. Text responses were trimmed, spelling variants were harmonized and categorical variables were converted into consistent response levels.

Age required additional cleaning because the source file contained numeric ages, birth years and date-like entries. Numeric ages within the prespecified plausible range were retained as reported. Values that appeared to represent years or dates of birth were converted to age using the response date. Source verification identified three data-entry errors: one recorded age of 16 years represented 26 years, and two recorded ages of 17 years represented 27 years. These values were corrected before age summaries and age-adjusted analyses. Values outside the prespecified range of 18 to 100 years after correction were treated as missing. All 495 respondents had a valid adult age after cleaning, with a range of 18 to 56 years.

Frequency-based practice responses were converted to ordered scores from 0 to 4, where 0 represented Never, 1 Rarely, 2 Sometimes, 3 Often and 4 Always. Symptom-recognition items were coded as selected or not selected.

Missing values were handled item by item for descriptive analyses. The final dataset contained complete information for the principal domain scores reported here; regression models used available complete data for the variables included in each model.

### 2.4 Survey instrument development and domains

The questionnaire used in this study was adapted from the structured household chemical indoor-pollution instrument reported by Montuori et al. (22). In that original study, the questionnaire was developed by a multidisciplinary group comprising physicians, chemists and biologists and was pilot-tested with 10 individuals to assess item comprehension. That pilot testing pertained to the original instrument and does not constitute Lebanon-specific validation.

The study materials available for this secondary analysis did not preserve sufficiently detailed item-level provenance to determine with certainty which items were retained verbatim, contextually modified or newly introduced for the Lebanon implementation. The available documentation also did not indicate a separate Lebanon-specific cognitive pilot, formal translation/back-translation procedure or external criterion validation. Accordingly, the scores and indices used here are treated as study-specific measures rather than as established psychometrically validated scales for the Lebanese population.

The analytical questionnaire comprised four domains. The knowledge domain contained 12 items assessing awareness of indoor chemical pollution sources and mechanisms, including gas stoves, poor ventilation, third-hand smoke, second-hand smoke, formaldehyde, sick-building syndrome, indoor-outdoor comparisons and household plants.

The preventive-attitude domain contained 12 items assessing respondents’ orientation toward behaviors that may reduce or increase indoor chemical exposure. Items addressed ventilation, cleaning, product selection, aerosol deodorants, candles, incense, gas appliances, decorative heating sources and smoking-related practices.

The household-practice domain contained 12 frequency items describing routine behavior in the home: ventilation, air-purifier use, kitchen-extractor use, HVAC filter use, HVAC inspection, washing curtains or carpets, gas-stove use, gas-heater or water-heater use, air-freshener use, pesticide use, ornamental plant keeping and indoor smoking.

The symptom-recognition domain asked respondents to identify possible warning signs associated with chemical indoor-air exposure. Items included respiratory, neurological, ocular, irritant, dermatological and gastrointestinal manifestations. For Figure 1 only, symptoms were also summarized into broad clinical domains as a descriptive visualization; these grouped domains were not used as inferential outcomes.

**Figure 1:**
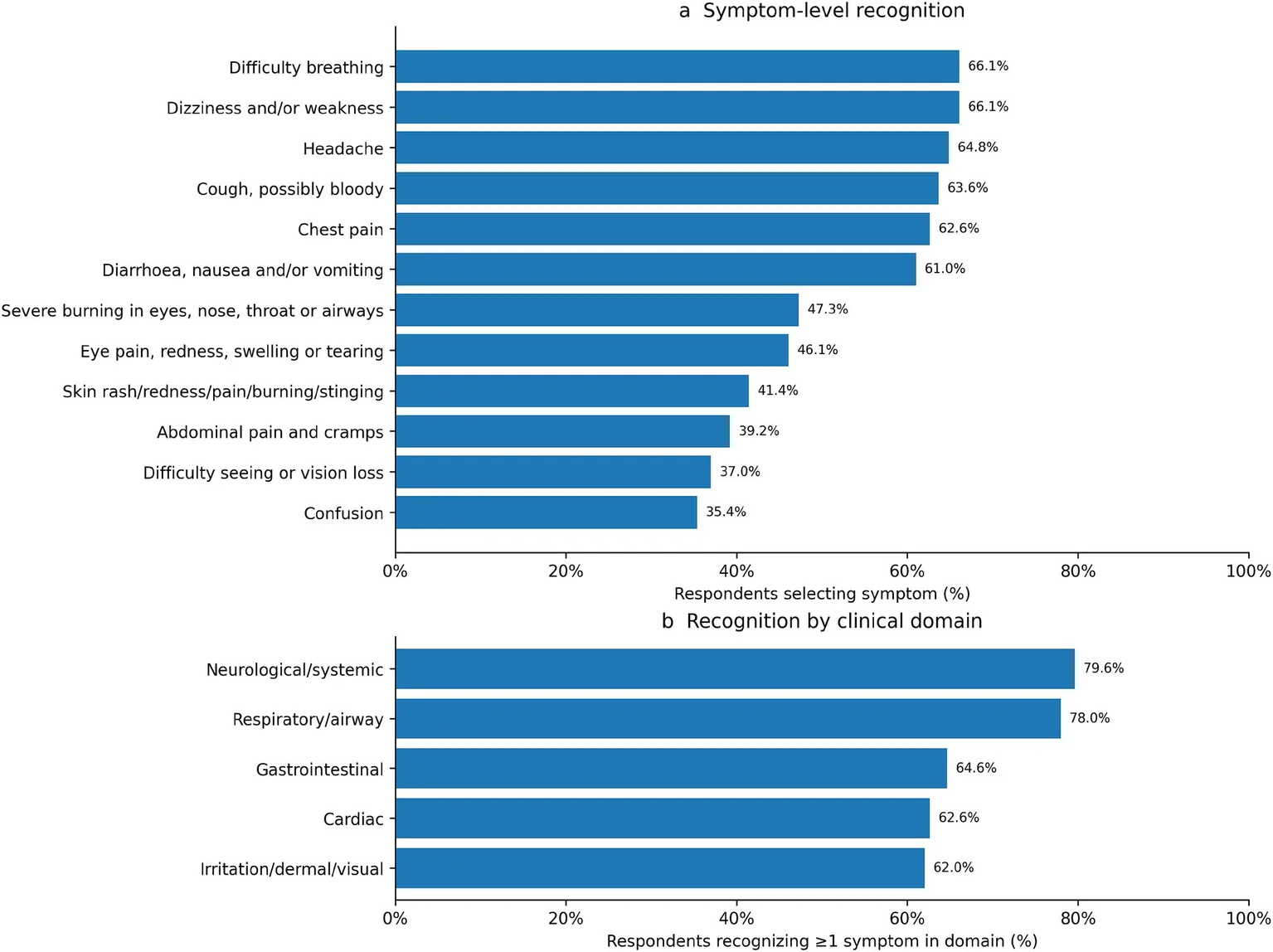
Symptom-recognition profile. Panel **(a)** shows item-level recognition of possible warning signs. Panel **(b)** summarizes the proportion recognizing at least one symptom within broad clinical domains for descriptive purposes. These measures assess recognition only and do not represent self-reported disease prevalence or clinically verified exposure outcomes.

### 2.5 Knowledge score

Knowledge items were scored using an *a priori* evidence-aligned answer key. Each item received one point when the respondent selected the response consistent with the study’s evidence-based scoring direction and a zero point when knowledge was not confirmed because the respondent selected an uncertain or non-aligned response. This binary score was used to quantify confirmed knowledge alignment; it does not imply that uncertainty is conceptually equivalent to a confidently incorrect belief. Item-level results therefore retain uncertainty as a separate response category.

Agreement was scored as aligned for statements indicating that gas stoves contribute to home pollution, poor ventilation contributes to indoor chemical pollution, formaldehyde can be a household pollutant, sick-building syndrome can be associated with indoor environmental conditions and third-hand smoke can persist as toxic residue indoors. Disagreement was scored as aligned for statements that minimized indoor exposure or residual tobacco-smoke risk. Comparative or context-dependent statements, particularly indoor-outdoor comparisons and relative second- versus third-hand smoke wording, are interpreted cautiously at item level and are not treated as stand-alone quantitative rankings of hazards.

The total knowledge score was calculated as the percentage of evidence-aligned responses across the 12 knowledge items and ranged from 0 to 100, with higher scores indicating greater confirmed evidence alignment within this study-specific instrument.

### 2.6 Preventive-attitude score

Preventive attitudes were scored using the same general principle but were interpreted as protective orientation rather than factual knowledge. Each response was classified according to whether it favored source control or exposure reduction within the prespecified scoring framework.

Agreement with routine ventilation and winter ventilation was coded as protective. Disagreement with statements normalizing potentially harmful or source-related behaviors, such as treating all cleaning products as equivalent, describing aerosol deodorants as healthy or treating candles, incense, decorative heaters and indoor smoking as harmless comfort practices, was also coded as protective.

The preventive-attitude score was calculated as the percentage of protective responses across the 12 attitude items and ranged from 0 to 100, with higher values indicating a stronger source-control and exposure-reduction orientation. The aggregate was retained as a study-specific summary, not as a validated measure of a single psychological construct. Its items may reflect several domains, including personal risk appraisal, perceived household norms and perceived control over source reduction, which the questionnaire was not designed to separate.

Some attitude statements involve context-dependent trade-offs. For example, opening windows while air conditioning operates may conflict with thermal comfort, energy use, outdoor dust or outdoor pollution. Such items were retained because they formed part of the prespecified questionnaire and scoring system, but their item-level interpretation is explicitly cautious.

### 2.7 Household practice indices

Household practices were analyzed using two separate study-specific indices: a mitigation index and a source-frequency index. The separation was intended to avoid treating all practices as a single safe-versus-unsafe continuum because protective and source-related activities can coexist within the same household.

The mitigation index summarized six behaviors intended to reduce or control indoor exposure: home ventilation, air-purifier use, kitchen-extractor use, HVAC filter use, periodic HVAC inspection and washing curtains or carpets.

The source-frequency index summarized five reported household activities that were classified *a priori* as source-related: gas oven or stove use, gas heater or water-heater use, air-freshener use, indoor pesticide use and indoor smoking. Ornamental plant keeping was described separately but excluded from this index because it does not have the same toxicological interpretation as combustion, tobacco smoke, pesticides or fragranced products. This correction aligns the Methods with the five-item index used for all reported source-frequency statistics and models.

Each practice item was scored from 0 to 4 according to reported frequency. Items were equally weighted to provide a transparent summary of reported frequency and to avoid imposing unsupported toxicity or exposure weights in the absence of measured concentrations, durations and activity-specific emission factors. The mean item score for each index was rescaled to 0–100. Higher mitigation scores indicate more frequent performance of selected exposure-reducing behaviors, whereas higher source-frequency scores indicate more frequent performance of the selected source-related activities. Equal numerical weighting does not imply equal emissions, toxicity, modifiability or health relevance, and alternative weighting schemes could change individual index values and observed associations. Neither index is a measured pollutant dose or exposure-burden metric.

An exploratory mitigation-minus-source difference score was calculated by subtracting the source-frequency index from the mitigation index. Positive values indicate that the selected mitigation practices were reported more frequently than the selected source-related practices, whereas negative values indicate the reverse. Because the two components represent heterogeneous behaviors with equal numerical weighting, this difference score is descriptive and should not be interpreted as physical exposure equivalence, risk compensation or evidence that one behavior cancels another.

### 2.8 Symptom-recognition count

The symptom-recognition item was analyzed as a public-health literacy measure rather than as a clinical outcome. Respondents were asked to identify symptoms that may occur with chemical indoor-air exposure, and each selected symptom was coded as recognized.

A symptom-recognition count was calculated by summing the number of symptoms selected. Higher counts indicate broader recognition of possible warning signs; the variable does not measure disease prevalence, poisoning events, actual exposure or medically confirmed outcomes.

### 2.9 Measurement properties and construct interpretation

Internal consistency was calculated for transparency using Cronbach’s alpha for the knowledge score, preventive-attitude score, mitigation index and source-frequency index. Alpha was interpreted cautiously because the household-practice indices combine distinct behaviors rather than interchangeable manifestations of a single latent trait.

Knowledge and preventive-attitude items were expected to show moderate internal coherence because they represent related cognitive and attitudinal constructs. The mitigation and source-frequency indices were instead treated primarily as formative behavioral summaries: ventilation, HVAC maintenance, cooking, fragrance use, pesticide use and smoking are noninterchangeable actions that jointly characterize reported household behavior. For formative indices, internal consistency is not an appropriate stand-alone validity criterion (27, 28).

Accordingly, the practice indices are not described as validated unidimensional scales. Their interpretation rests on content relevance, transparent item composition and expected associations with knowledge and preventive attitudes. No external validation against observed household behavior, building characteristics or measured pollutant concentrations was available.

### 2.10 Statistical analyses

Descriptive statistics summarized participant characteristics and domain scores. Categorical variables are presented as counts and percentages; continuous scores are summarized using means, standard deviations, medians and interquartile ranges. Wilson 95% confidence intervals were calculated for selected binomial proportions. Because the sample was non-probability and unweighted, these intervals express statistical precision under the analytic assumptions rather than design-based national population uncertainty.

Item-level results were presented for knowledge, preventive attitudes, household practices and symptom recognition. Knowledge and attitude items are summarized using evidence-aligned or protective responses together with uncertainty. Practice items are summarized across the original frequency categories and interpreted within their mitigation or source-related grouping.

Spearman rank correlations were used to examine monotonic associations among knowledge score, preventive-attitude score, mitigation index, source-frequency index, mitigation-minus-source difference score and symptom-recognition count. Bootstrap resampling was used to estimate confidence intervals for selected correlation coefficients.

Multivariable linear regression models were fitted for four continuous outcomes: mitigation index, source-frequency index, mitigation-minus-source difference score and symptom-recognition count. Heteroskedasticity-robust HC3 standard errors were used for these models.

The principal predictors were knowledge score per 10-point increase, preventive-attitude score per 10-point increase, age per 10-year increase, gender, current smoking status, presence of children, university-level education and region group.

Logistic regression models with robust inference were also fitted for two binary behavioral outcomes: regular indoor smoking and frequent kitchen-extractor use. Regular indoor smoking was defined as smoking inside the home often or always, and frequent kitchen-extractor use was defined as using the kitchen hood or extractor often or always. Gas-stove frequency was included as an additional covariate in the extractor model because extractor use is directly related to cooking behavior.

Linear-model estimates are reported as unstandardized adjusted coefficients (*B*) with 95% confidence intervals and *p*-values; logistic models are reported as adjusted odds ratios (ORs). All *p*-values were two-sided and interpreted as exploratory rather than confirmatory.

### 2.11 Sensitivity analysis

A sensitivity analysis examined whether the association between knowledge and mitigation behavior depended on potentially ambiguous knowledge items. The knowledge score was recalculated after excluding the poor-ventilation and regulatory-guidance items, which may partly reflect general policy or ventilation awareness rather than source-specific indoor-air chemistry.

The mitigation model was then re-estimated using the revised knowledge score. This analysis was used as a robustness check on the principal interpretation of the knowledge-mitigation relationship.

Because the study was cross-sectional and exploratory, statistical inference was used to identify patterns that may inform future research and intervention design rather than to establish causal effects. Multiple-testing correction was not applied; effect sizes, confidence intervals and consistency across analyses were emphasized over isolated *p*-values.

### 2.12 Software and reproducibility

Data cleaning, scoring, statistical analysis and figure generation were performed using Python version 3.14.6. Data manipulation used pandas version 3.0.3 and NumPy version 2.5.0; statistical analyses used SciPy version 1.18.0 and statsmodels version 0.14.6; figures were generated using Matplotlib version 3.11.0. The analysis workflow was implemented using reproducible Python scripts.

### 2.13 Ethical considerations

The study was approved by the Institutional Review Board of Canadian University Dubai and was conducted in accordance with the Declaration of Helsinki. The first page of the online survey contained written informed-consent information describing the target population, study purpose, potential risks and benefits and confidentiality protections. Participants were informed that participation was voluntary and that submission of the questionnaire indicated consent to participate.

## 3 Results

Participant profile and domain scores. The analytic sample included 495 respondents, with a median age of 22 years (IQR 20–24; range 18–56) after correction of three verified age-entry errors. Females represented 77.6% of the sample. Beirut accounted for 36.6% of respondents and Mount Lebanon for 25.7%, with the remaining respondents distributed across the other governorates (Table 1 and Figure 2). The sample was highly educated: 90.3% reported university education and 5.7% reported a master’s degree. Current smoking was reported by 25.9%, 12.9% reported children in the household and 3.4% reported previous hospitalization because of chemical poisoning. These descriptive values characterize the convenience sample and should not be interpreted as nationally representative estimates for Lebanon.

**Table 1:** Participant characteristics and domain-score summary for the analytic convenience sample (*n* = 495).

Characteristic	Estimate	Additional statistic
Total respondents	495	–
Age, median (IQR)	22 (20–24)	Range 18–56
Gender		
Female	384 (77.6%)	–
Male	111 (22.4%)	–
Governorate		
Beirut	181 (36.6%)	–
Mount Lebanon	127 (25.7%)	–
South Lebanon	60 (12.1%)	–
North Lebanon	39 (7.9%)	–
Nabatieh	26 (5.3%)	–
Akkar	25 (5.1%)	–
Baalbek-Hermel	19 (3.8%)	–
Bekaa	18 (3.6%)	–
Education		
University	447 (90.3%)	–
Master’s degree	28 (5.7%)	–
Secondary	9 (1.8%)	–
Doctorate	9 (1.8%)	–
Primary	1 (0.2%)	–
Intermediate	1 (0.2%)	–
Current smoker: Yes	128 (25.9%)	–
Children in household: Yes	64 (12.9%)	–
Prior hospitalization for chemical poisoning: Yes	17 (3.4%)	–
Knowledge score (0–100)	47.3 ± 22.3	Median 50.0 (IQR 33.3–66.7)
Preventive-attitude score (0–100)	48.8 ± 22.8	Median 50.0 (IQR 33.3–66.7)
Mitigation index (0–100)	66.3 ± 18.9	Median 66.7 (IQR 54.2–79.2)
Source-frequency index (0–100)	58.4 ± 19.8	Median 60.0 (IQR 45.0–70.0)
Mitigation-minus-source difference	7.9 ± 20.6	Median 6.7 (IQR − 5.0-20.0)

**Figure 2:**
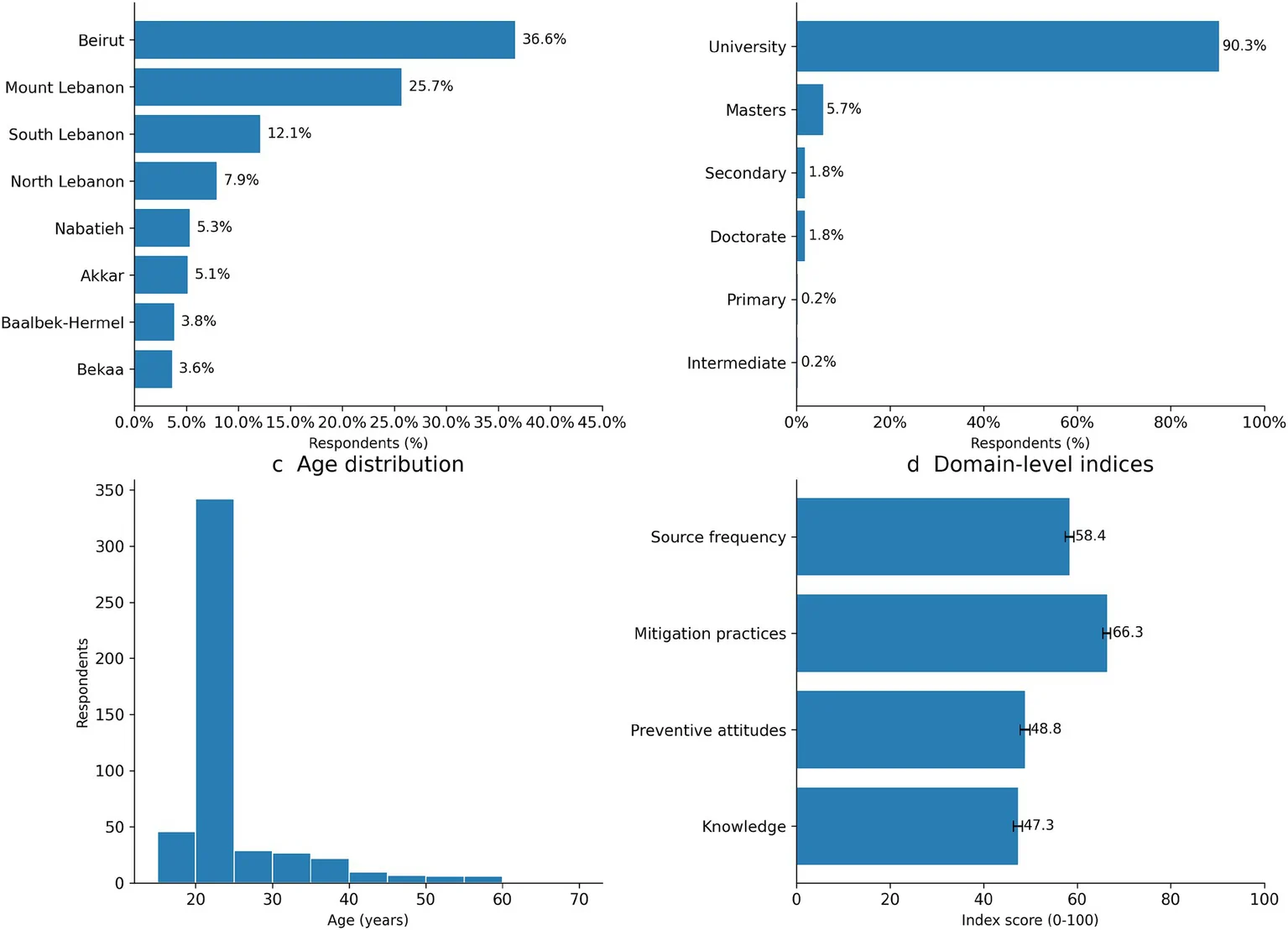
Sample profile and analytic domain scores. Panel **(a)** shows governorate distribution; panel **(b)** educational attainment; panel **(c)** age distribution; and panel **(d)** mean 0-100 scores for knowledge, preventive attitudes, mitigation practices and source-frequency practices. Values are unweighted and describe the analytic sample rather than the Lebanese population.

The mean knowledge score was 47.3 of 100 (SD 22.3), and the mean preventive-attitude score was 48.8 (SD 22.8). The mitigation index was higher at 66.3 (SD 18.9), while the source-frequency index averaged 58.4 (SD 19.8). The exploratory mitigation-minus-source difference score averaged 7.9 points (SD 20.6). Figure 2 summarizes the sample profile and the four principal 0–100 domain scores. The pattern indicates that frequent mitigation practices and frequent source-related activities can coexist; the practice indices should therefore be interpreted as complementary behavioral summaries rather than as a single measure of exposure or safety.

Internal consistency was moderate for the knowledge score (Cronbach’s alpha = 0.696) and preventive-attitude score (alpha = 0.738), lower for the mitigation index (alpha = 0.667) and lower still for the five-item source-frequency index (alpha = 0.556). For the source-frequency items, corrected item-total correlations ranged from 0.223 to 0.410 and pairwise inter-item correlations ranged from 0.076 to 0.396. All correlations were positive; gas oven or stove use had the lowest corrected item-total correlation (0.223), consistent with the heterogeneous formative interpretation rather than indicating a negatively functioning item. These values reinforce that the practice indices are behavioral summaries rather than unidimensional psychological scales.

Knowledge profile and evidence-specific uncertainty. Respondents recognized several familiar sources and mechanisms. Agreement that second-hand smoke results from active smoking was 74.1%, agreement that gas ovens or stoves contribute to home pollution was 66.9%, agreement that poor ventilation contributes to chemical indoor pollution was 64.8 and 63.2% recognized third-hand smoke as toxic residue in the indoor environment (Table 2 and Figure 3). Uncertainty was greater for less visible or more conceptually difficult items. Only 17.6% selected disagreement with the comparative statement that third-hand smoke is less hazardous than second-hand smoke, while 46.3% were unsure. Because relative hazard depends on exposure context, this item is interpreted as uncertainty about residual tobacco-smoke risk rather than as a validated quantitative ranking of second- versus third-hand smoke toxicity. Similarly, only 22.6% disagreed with the statement that indoor chemical pollution is lower than outdoor pollution. Indoor-outdoor relationships vary by pollutant, source strength, infiltration and ventilation, so this item is interpreted as awareness that indoor exposures can be substantial rather than as a universal claim that indoor concentrations exceed outdoor concentrations. Formaldehyde and sick-building-syndrome items also showed high uncertainty.

**Table 2:** Knowledge items highlighting priority uncertainty and selected source concepts.

Knowledge item	Evidence-aligned response	Evidence-aligned (%)	Unsure (%)
Third-hand smoke is less hazardous than second-hand smoke	Disagree	17.6%	46.3%
Plants release dangerous materials at night	Disagree	18.2%	33.5%
Indoor chemical pollution is lower than outdoor pollution	Disagree	22.6%	28.3%
Third-hand smoke is toxic residue in the indoor environment	Agree	63.2%	32.9%
Gas ovens/stoves contribute to home pollution	Agree	66.9%	29.1%
Poor ventilation strongly contributes to chemical indoor pollution	Agree	64.8%	32.5%

**Figure 3:**
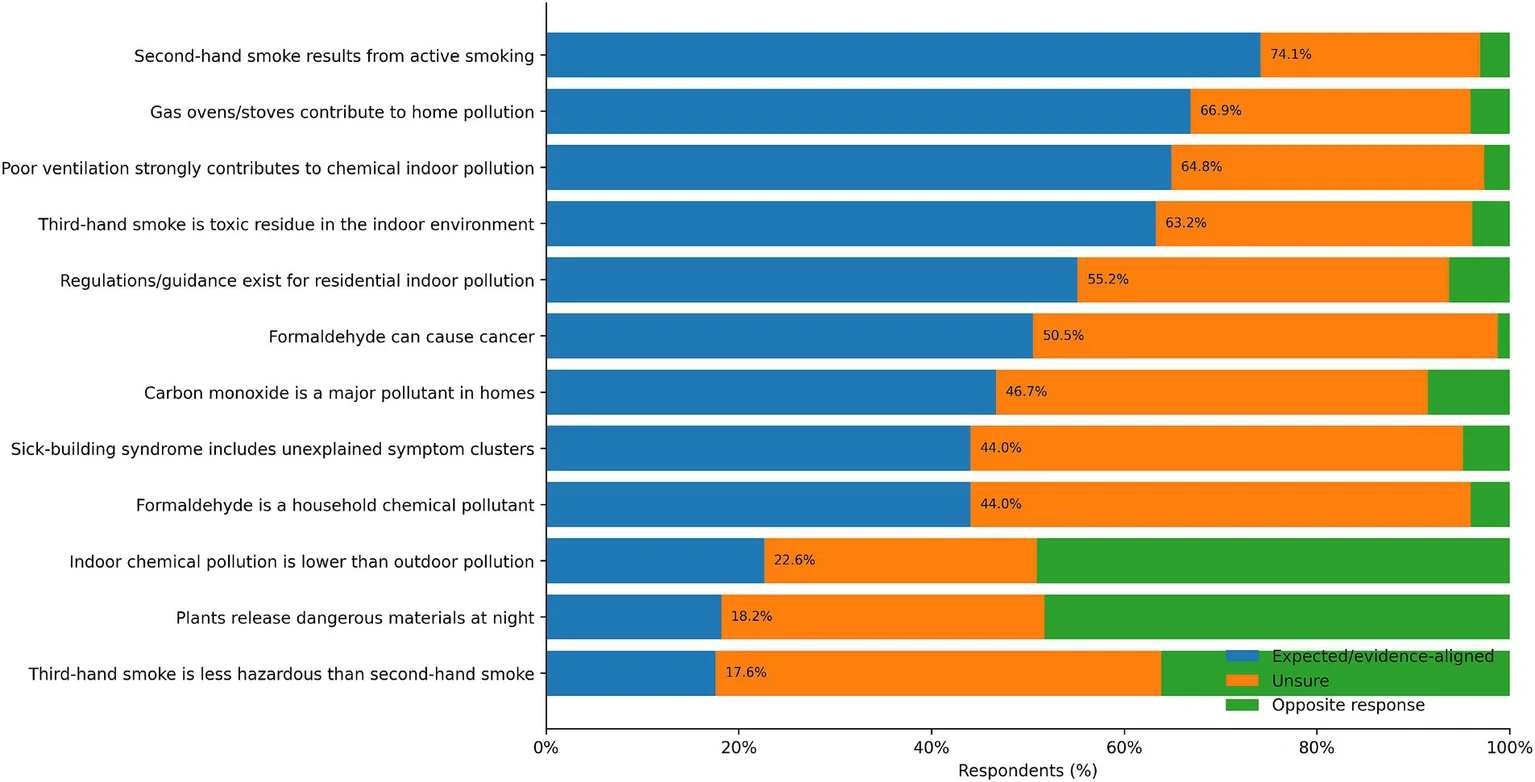
Knowledge profile for residential chemical indoor-air pollution. Bars show the proportions selecting the prespecified evidence-aligned response, an unsure response or the opposite response for each item. Comparative and context-dependent items are interpreted cautiously rather than as stand-alone measures of quantitative hazard ranking.

The most actionable knowledge deficits concern mechanisms that are difficult to detect without targeted education. Third-hand smoke involves persistent surface and dust reservoirs that can continue to expose occupants after active smoking stops (16, 17). Formaldehyde can arise from building materials, furnishings, combustion and secondary indoor chemistry (18, 19). The household-plant item is retained as a belief item from the survey but is not treated as a major chemical-hazard marker. Overall, the item-level results point to a need for communication about invisible sources, residual exposure and the context dependence of indoor-outdoor pollution rather than simple binary messages.

Preventive attitudes showed a parallel contrast between broad protective norms and more context-dependent comfort or product practices (Table 3). Most respondents agreed that opening windows is a good habit (81.0%) and that winter ventilation several times per day is necessary (78.4%). Protective responses were less frequent for aerosol deodorants, candles and incense. The air-conditioning/window item was especially uncertain: 20.2% gave the prespecified protective response and 40.8% were unsure. Because ventilation while cooling is context dependent in relation to heat, outdoor dust, outdoor pollution and energy use, this item is interpreted cautiously and is not used alone to characterize appropriate ventilation behavior.

**Table 3:** Preventive-attitude items highlighting comfort- and product-related uncertainty.

Attitude item	Protective response	Protective (%)	Unsure (%)
Opening windows while air conditioning runs is necessary	Disagree	20.2%	40.8%
Aerosol deodorant use is healthy	Disagree	35.8%	42.8%
All cleaning products are good without difference	Disagree	63.8%	25.9%
Opening windows is a good habit	Agree	81.0%	16.4%
Winter ventilation several times/day is necessary	Agree	78.4%	17.6%

The attitude profile is consistent with evidence that fragranced products, candles and incense can emit volatile organic compounds, particles and reactive constituents, although actual exposure depends on product composition, duration, room size and ventilation (11, 12, 14, 29). The findings therefore support source-specific communication rather than blanket judgments: residents need information about which activities can increase emissions, how intensity and duration matter and how source control complements ventilation.

Household practice landscape. Figure 4 and Table 4 show that home ventilation was the dominant reported mitigation practice: 88.7% ventilated often or always and 99.0% reported at least some ventilation. Washing curtains or carpets was also frequent, with 82.2% reporting often or always. More device- or maintenance-dependent practices were less frequent: 53.1% often or always used a kitchen extractor, 52.3% used HVAC filters, 51.1% reported periodic HVAC inspection and 40.2% used an air purifier. These results suggest that familiar low-cost actions were more embedded than equipment-dependent behaviors, although the survey did not assess effectiveness, device specifications or actual ventilation rates.

**Figure 4:**
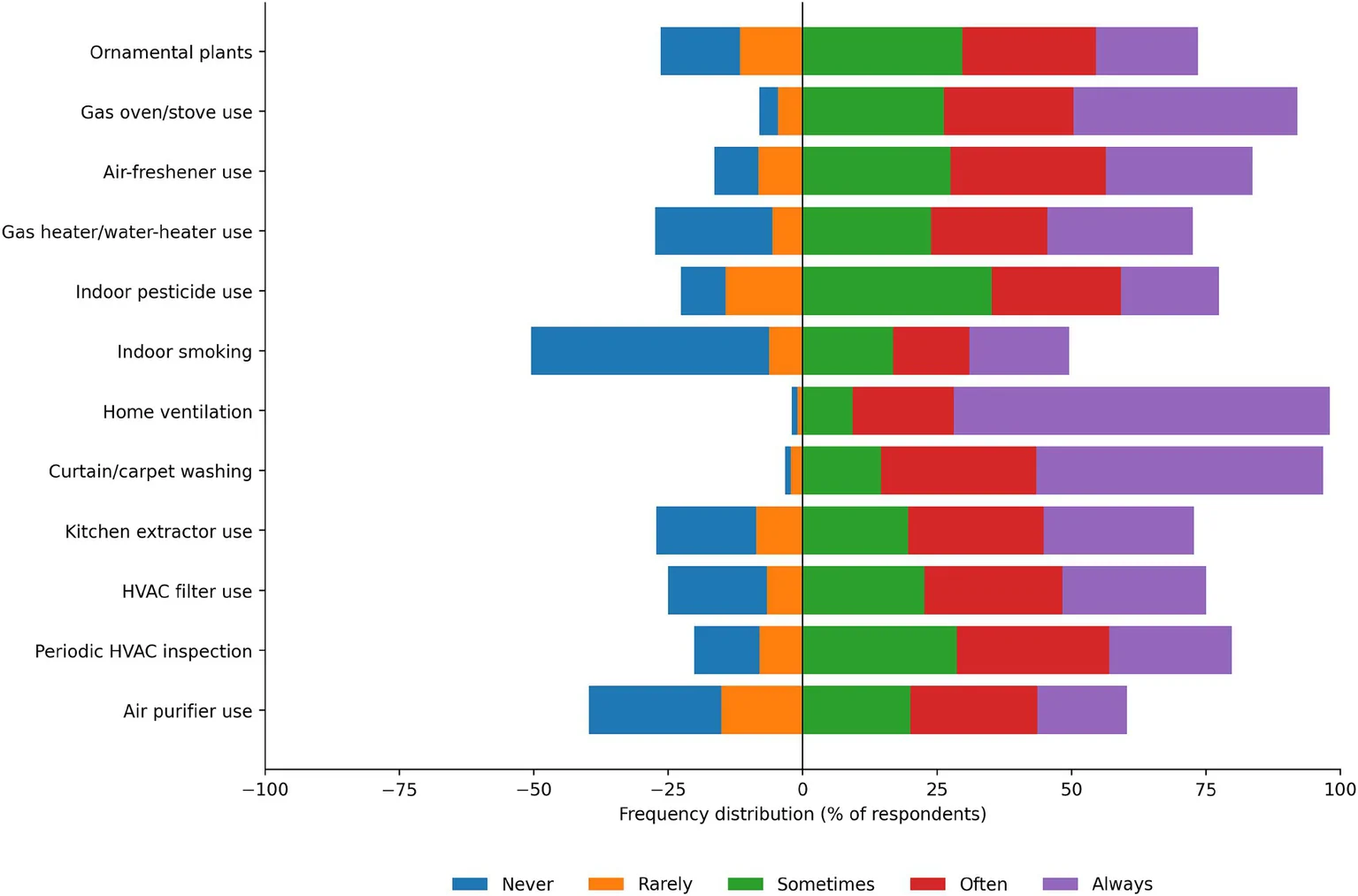
Household practice landscape. Frequency distributions are shown for mitigation and source-related household activities. Ornamental plant keeping is displayed descriptively because it was included in the questionnaire, but it was excluded from the source-frequency index and should not be interpreted as toxicologically equivalent to combustion, tobacco smoke, pesticides or fragranced products.

**Table 4:** Frequency of selected mitigation and source-related household practices.

Practice	Domain	Often/always	Any use
Home ventilation	Mitigation	88.7%	99.0%
Curtain/carpet washing	Mitigation	82.2%	99.0%
Gas oven/stove use	Source-related	65.7%	96.6%
Air-freshener use	Source-related	56.2%	91.9%
Kitchen extractor use	Mitigation	53.1%	81.4%
HVAC filter use	Mitigation	52.3%	81.6%
Periodic HVAC inspection	Mitigation	51.1%	87.9%
Gas heater/water-heater use	Source-related	48.7%	78.2%
Indoor pesticide use	Source-related	42.2%	91.7%
Air purifier use	Mitigation	40.2%	75.4%
Indoor smoking	Source-related	32.7%	55.8%

Source-related household activities were also common. Gas oven or stove use was reported often or always by 65.7% of respondents and any use by 96.6%. Gas heater or water-heater use was reported often or always by 48.7%, air-freshener use by 56.2%, indoor pesticide use by 42.2% and indoor smoking by 32.7%. The coexistence of frequent mitigation with frequent source-related activity is central to the study: ventilation and filtration may reduce concentrations but are not equivalent to eliminating an emission source. Gas cooking and heating can emit nitrogen dioxide, carbon monoxide, formaldehyde and benzene under residential conditions (4–6), while air fresheners and pesticides introduce additional volatile or semi-volatile chemicals. The source-frequency index nevertheless remains a behavioral frequency measure rather than an exposure-dose metric.

Symptom recognition. Respondents most frequently recognized difficulty breathing and dizziness or weakness (both 66.1%), headache (64.8%), cough possibly bloody (63.6%), chest pain (62.6%) and gastrointestinal symptoms (61.0%) as possible manifestations of chemical exposure (Table 5 and Figure 1). Recognition was lower for severe burning in the eyes, nose, throat or airways (47.3%), eye pain or redness (46.1%), skin manifestations (41.4%), abdominal pain or cramps (39.2%), difficulty seeing or vision loss (37.0%) and confusion (35.4%). These results describe warning-sign literacy, not the prevalence of symptoms or disease among respondents.

**Table 5:** Recognition of possible symptoms associated with chemical indoor-air exposure.

Recognized symptom	Recognized	95% CI
Difficulty breathing	66.1%	61.8 to 70.1%
Dizziness and/or weakness	66.1%	61.8 to 70.1%
Headache	64.8%	60.5 to 68.9%
Cough, possibly bloody	63.6%	59.3 to 67.8%
Chest pain	62.6%	58.3 to 66.8%
Diarrhoea, nausea and/or vomiting	61.0%	56.6 to 65.2%
Severe burning in eyes, nose, throat or airways	47.3%	42.9 to 51.7%
Eye pain, redness, swelling or tearing	46.1%	41.7 to 50.5%
Skin rash/redness/pain/burning/stinging	41.4%	37.2 to 45.8%
Abdominal pain and cramps	39.2%	35.0 to 43.6%
Difficulty seeing or vision loss	37.0%	32.8 to 41.3%
Confusion	35.4%	31.3 to 39.7%

Associations among knowledge, attitudes, practices and symptom recognition. Knowledge and preventive-attitude scores were moderately correlated (Spearman rho = 0.352, 95% CI 0.269 to 0.428, *p* < 0.001), supporting an association between evidence alignment and protective orientation. Knowledge showed only weak correlation with the mitigation index (rho = 0.077, 95% CI −0.019 to 0.167, *p* = 0.088) and with the source-frequency index (rho = −0.039, *p* = 0.391). Preventive attitudes were inversely correlated with source-frequency practices (rho = −0.233, 95% CI −0.315 to −0.142, *p* < 0.001). The mitigation and source-frequency indices were positively correlated (rho = 0.410, 95% CI 0.329 to 0.485, *p* < 0.001), demonstrating that respondents who reported more household management activity often reported more of both mitigation and source-related practices rather than falling into distinct safe and unsafe groups.

Multivariable models refined this interpretation (Table 6 and Figure 5). In the source-frequency model, each 10-point increase in preventive-attitude score was associated with a 1.58-point lower source-frequency index (95% CI −2.37 to −0.79, *p* < 0.001) after adjustment for knowledge, corrected age, gender, smoking, children, education and region group. Current smoking was associated with a 13.26-point higher source-frequency index (95% CI 9.55 to 16.97, *p* < 0.001); because indoor smoking is itself one component of the source-frequency index, this coefficient partly reflects expected item overlap and is interpreted descriptively rather than as an independent etiologic effect. For the mitigation-minus-source difference score, each 10-point increase in preventive attitude was associated with a 1.41-point higher difference (95% CI 0.60 to 2.22, *p* < 0.001). Knowledge was not independently associated with the mitigation index after adjustment (*B* = 0.72 per 10 points, 95% CI −0.15 to 1.58, *p* = 0.103).

**Table 6:** Selected adjusted associations from robust regression models.

Outcome	Predictor	Effect measure	Estimate	95% CI	*p*
Source-frequency index	Preventive attitude score, per 10 points	Adjusted B	−1.58	−2.37 to −0.79	<0.001
Source-frequency index	Current smoker	Adjusted B	13.3	9.55 to 17.0	<0.001
Mitigation-minus-source difference	Preventive attitude score, per 10 points	Adjusted B	1.41	0.60 to 2.22	<0.001
Mitigation-minus-source difference	Current smoker	Adjusted B	−10.1	−14.3 to −5.98	<0.001
Mitigation index	Knowledge score, per 10 points	Adjusted B	0.72	−0.15 to 1.58	0.103
Mitigation index	Preventive attitude score, per 10 points	Adjusted B	−0.17	−0.98 to 0.64	0.679
Recognized symptom count	Knowledge score, per 10 points	Adjusted B	0.41	0.24 to 0.58	<0.001
Recognized symptom count	Preventive attitude score, per 10 points	Adjusted B	0.25	0.09 to 0.40	0.002
Recognized symptom count	Age, per 10 years	Adjusted B	0.91	0.18 to 1.64	0.015
Regular indoor smoking	Preventive attitude score, per 10 points	Adjusted OR	0.77	0.69 to 0.87	<0.001
Regular indoor smoking	Current smoker	Adjusted OR	13.2	7.57 to 23.0	<0.001
Kitchen hood often/always	Knowledge score, per 10 points	Adjusted OR	1.11	1.01 to 1.22	0.023

**Figure 5:**
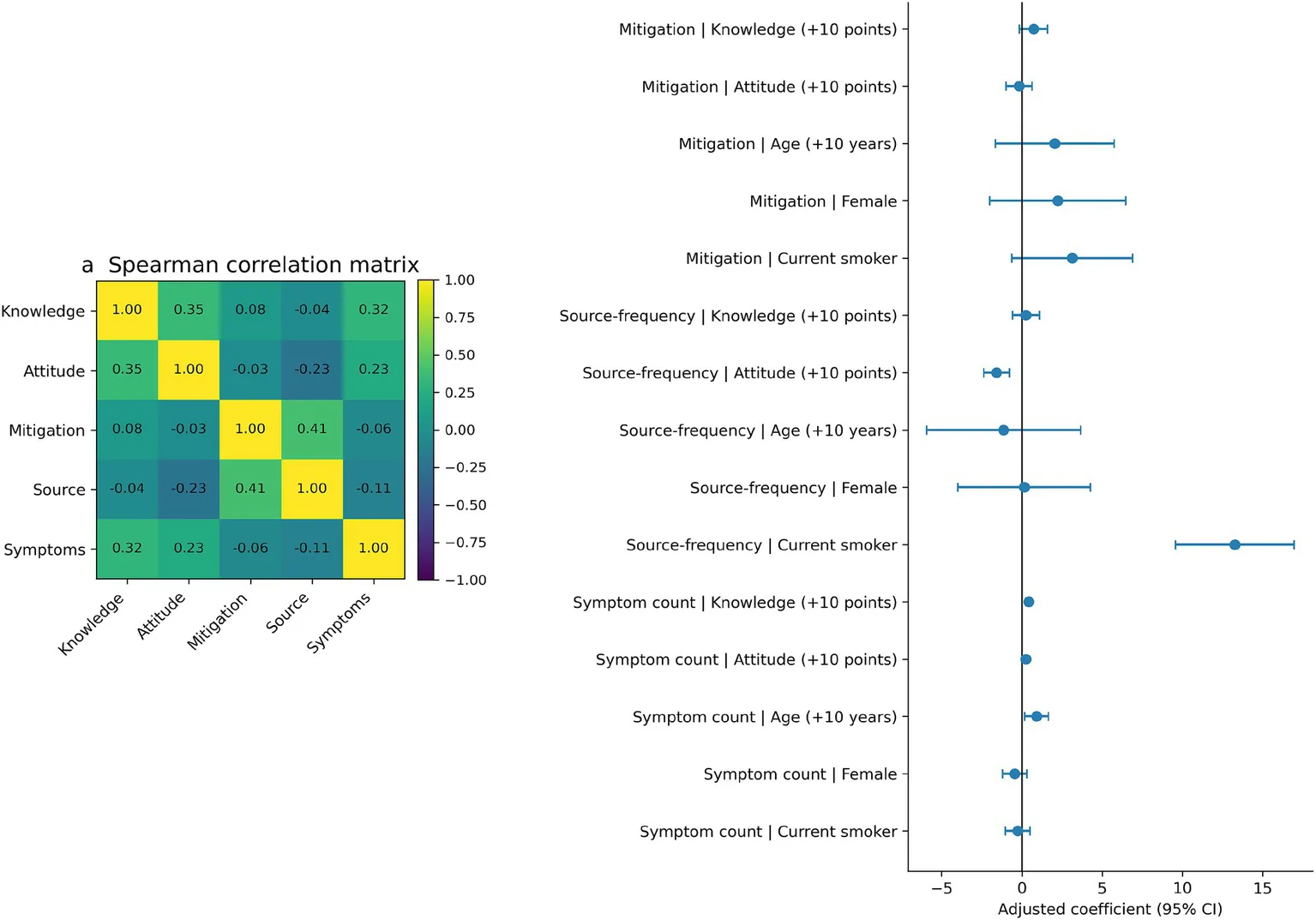
Knowledge-practice decoupling and adjusted correlates. Panel **(a)** shows the Spearman correlation matrix among knowledge, preventive attitudes, mitigation, source-frequency and symptom-recognition scores. Panel **(b)** shows selected adjusted coefficients and 95% confidence intervals from robust linear models for continuous outcomes. The figure is descriptive of associations and does not imply causality.

The logistic models provided additional source-specific information. Each 10-point increase in preventive-attitude score was associated with lower odds of regular indoor smoking (adjusted OR 0.77, 95% CI 0.69 to 0.87, *p* < 0.001). Current smoking status was, as expected, strongly associated with regular indoor smoking (adjusted OR 13.20, 95% CI 7.57 to 23.03, *p* < 0.001) and should be viewed primarily as an expected-group check rather than a novel behavioral finding. For frequent kitchen-extractor use, knowledge showed a small positive association (adjusted OR 1.11 per 10 points, 95% CI 1.01 to 1.22, *p* = 0.023), while preventive-attitude score was not associated. In the symptom-recognition model, knowledge and preventive attitudes were both positively associated with the number of recognized symptoms; corrected age was also positively associated (*B* = 0.91 per 10 years, 95% CI 0.18 to 1.64, *p* = 0.015). These cross-sectional associations do not establish that increasing knowledge or attitudes would cause behavior change.

## 4 Discussion

### 4.1 Principal findings

This study identified a clear separation between what respondents knew or endorsed and what they reported doing at home. Familiar concepts such as second-hand smoke, gas stoves and poor ventilation were recognized relatively well, whereas uncertainty was greater for less visible mechanisms, including formaldehyde, sick-building syndrome and residual tobacco-smoke exposure. At the same time, mitigation practices were common but coexisted with frequent gas-stove use, air-freshener use, pesticide use and indoor smoking. The resulting pattern is better described as partial knowledge embedded in routine household practices than as simple ignorance.

The separation of mitigation and source-frequency practices is analytically useful because indoor concentrations depend on both emissions and removal processes. A respondent who ventilates daily may still use gas appliances, air fresheners, pesticides or tobacco indoors. Ventilation is therefore not evidence of low exposure; it is one control process operating alongside sources, deposition, filtration and indoor chemistry (2, 9, 30). The exploratory mitigation-minus-source difference score provides a compact descriptive summary of this coexistence but should not be interpreted as an exposure-equivalence or risk-compensation metric.

### 4.2 Knowledge and risk communication

Third-hand smoke illustrates the challenge of invisible persistence. Many respondents recognized that third-hand smoke leaves toxic residue, yet the comparative third-hand-versus-second-hand item produced substantial uncertainty. The scientifically robust message is therefore not a simple ranking of the two exposure types, but that tobacco residues can persist on surfaces and dust, undergo chemical transformation and contribute to exposure after active smoking has stopped (15–17). Household communication should emphasize smoke-free indoor rules and residue prevention rather than relying on visible smoke or odor as the indicator of risk.

Formaldehyde and sick-building-syndrome items point to a second communication challenge: technical indoor-air vocabulary may be unfamiliar even in a highly educated sample. Formaldehyde can originate from composite wood, furnishings, combustion and secondary indoor chemistry (18, 19). Educational materials should therefore connect technical terms with recognizable household sources and practical actions rather than assume familiarity with pollutant names. The indoor-versus-outdoor item should similarly be framed contextually: indoor exposure can be substantial for some pollutants and activities, but the relative indoor-outdoor relationship varies by pollutant, source strength, outdoor concentrations and ventilation.

### 4.3 Household practices and source control

Ventilation advice appears to have diffused into reported behavior, but source-control practices have not diffused with equal strength. Frequent home ventilation was nearly universal in the sample, whereas device-dependent mitigation such as extractor use, HVAC maintenance and air-purifier use was less consistent. In warm climates, simple window-opening advice can conflict with heat, humidity, outdoor pollution, dust, noise, security and energy costs. More useful guidance is source- and timing-specific: use effective local exhaust during cooking, maintain HVAC systems, ventilate during and after high-emission activities when outdoor conditions allow, reduce unnecessary fragranced products and maintain smoke-free indoor environments.

Gas-stove use is a high-priority example because it was common while frequent kitchen-extractor use was only moderate. Residential studies show that gas and propane stoves can increase nitrogen dioxide and benzene indoors and that exposure can extend beyond the kitchen (4–6). Knowledge showed a small adjusted association with frequent extractor use, suggesting that source-specific factual understanding may be relevant to this particular technical behavior. Future interventions should test concise cooking-ventilation messages and, where feasible, evaluate cleaner appliance alternatives while acknowledging affordability and infrastructure constraints.

Fragranced products, candles and incense require a different communication strategy because they may be intentionally used for scent, comfort or cultural reasons. Air-freshener use was frequent, and protective attitudes toward aerosol deodorants, candles and incense were limited. These products can emit volatile compounds, particles and reactive constituents, particularly in poorly ventilated rooms (9–12, 29). A practical public-health message is exposure minimization rather than blanket condemnation: reduce unnecessary frequency and duration, avoid simultaneous use of multiple scented products and improve ventilation after high-emission activities when conditions permit.

### 4.4 Behavioral interpretation

Knowledge was associated with preventive attitudes, but knowledge alone was only weakly related to mitigation behavior. Preventive attitudes showed a clearer inverse association with source-frequency and a positive association with the mitigation-minus-source difference score. Integrated behavioral frameworks help locate this gap more precisely. PADM-TPB pathways distinguish risk appraisal and attitudes from perceived norms, perceived behavioral control, intention and implementation; evidence from PM2.5 preventive behavior shows that awareness does not necessarily progress to adoption when these intermediate pathways are weak or constrained (20). The present questionnaire did not measure behavioral intention, perceived control or implementation barriers as distinct constructs. It therefore cannot determine whether the observed knowledge-practice decoupling reflects limited motivation, an intention-action gap, practical constraints or a combination of these mechanisms. The pattern remains consistent with broader knowledge-attitude-behavior research showing that factual information alone may be insufficient when convenience, perceived benefits, norms, cost and household routines shape action (21, 22, 24). Because the design is cross-sectional, the direction of these relationships cannot be established; stronger attitudes may influence practices, practices may reinforce attitudes or both may reflect unmeasured household characteristics.

Smoking-related findings should be interpreted with particular care. Current smokers had substantially higher source-frequency scores and much higher odds of regular indoor smoking, but indoor smoking is itself part of the source-frequency index and the strong association with regular indoor smoking is expected by definition. The more informative finding is that higher preventive-attitude scores were associated with lower odds of regular indoor smoking. This supports the relevance of household norms and source-specific risk perception while avoiding overinterpretation of the current-smoker coefficients.

### 4.5 Symptom-recognition literacy

Respiratory and neurological manifestations were recognized more often than ocular, irritant, dermatological and confusion-related signs. The survey did not ask whether respondents had experienced these symptoms, and the results therefore cannot estimate disease burden or identify poisoning events. The public-health implication is limited but useful: educational materials can broaden recognition of possible warning signs while making clear that nonspecific symptoms require clinical context and do not by themselves establish chemical exposure.

### 4.6 Local relevance and implications

Prior Lebanese research has demonstrated professional interest in air-pollution health effects, while regional and international studies show that knowledge, attitudes and behavior do not necessarily move together (21–23, 25). The present analysis adds a source-specific household perspective for Lebanon. Candidate communication priorities include smoke-free indoor rules, cooking extraction, reduced unnecessary fragranced-product use, safer cleaning and pesticide practices, formaldehyde literacy, HVAC maintenance and clearer explanation of invisible or persistent exposure mechanisms.

These findings are not sufficient to set national policy priorities because the sample was non-probability and demographically concentrated. They can, however, inform messages to be tested in representative studies. Household guidance should use plain language, distinguish odor masking from pollution reduction, connect specific sources with specific controls and acknowledge constraints related to climate, housing, cost and family norms. Clinicians, pharmacists, school health staff, community organizations and building professionals may all have roles in source-specific education, but intervention effectiveness should be evaluated rather than assumed.

### 4.7 Strengths and limitations

The study combines item-level knowledge, preventive attitudes, household practices and symptom-recognition literacy in one analytical framework and deliberately separates mitigation from source-frequency behavior. Several limitations nevertheless constrain interpretation. The sample was young, highly educated and predominantly female; the recruitment denominator and detailed channel-specific recruitment log were unavailable to the secondary analysis, so selection and nonresponse bias may be substantial. The survey was self-reported and cross-sectional, creating potential recall, social-desirability and common-method bias. The questionnaire was adapted from a previously published instrument, but Lebanon-specific cognitive testing, translation/back-translation documentation and external validation were not available in the secondary-analysis materials. Some items are context dependent, including indoor-outdoor comparisons and ventilation while air conditioning operates. Behavioral intention, perceived behavioral control and implementation barriers were not measured, so the analysis cannot distinguish failure to form a protective intention from failure to enact an existing intention. The preventive-attitude score also aggregates items that may reflect personal risk appraisal, household norms and perceived control; this limits construct-specific interpretation and may conceal different relationships between each dimension and behavior. The practice indices equally weight heterogeneous behaviors to summarize reported frequency and do not measure pollutant dose; alternative toxicological, emission-based or modifiability weights could yield different patterns. Ornamental plant keeping was excluded from the five-item source-frequency index because it does not have the same toxicological interpretation as combustion, smoking, pesticides or fragrance sources. The current-smoking predictor also overlaps conceptually with the indoor-smoking component of the source-frequency index. Cronbach’s alpha was modest for the practice indices, which is expected for formative behavioral summaries but reinforces that they should not be treated as unidimensional scales. Finally, symptom recognition is not a clinical endpoint and cannot be used to infer poisoning events or disease prevalence.

Future research should pair household questionnaires with objective environmental and behavioral measurements. Nitrogen dioxide, particulate matter, carbon dioxide and volatile-organic-compound monitoring, together with time-activity diaries, product-use inventories, building characteristics and ventilation measurements, would allow validation of the practice indices against exposure proxies. Future instruments should separately measure risk appraisal, perceived norms, perceived behavioral control, behavioral intention and implementation barriers, then test whether these dimensions explain adoption of specific protective actions. Sensitivity analyses using alternative source weights should also be compared with the transparent equal-frequency index. Longitudinal and intervention designs could test whether source-specific messages about gas cooking, third-hand smoke and fragranced products change household routines. Representative sampling across Lebanese governorates would also clarify how the patterns observed here vary by age, education, income, housing type, urbanicity and household composition.

## 5 Conclusion

This secondary cross-sectional analysis of a non-probability convenience sample in Lebanon found partial evidence-aligned knowledge, substantial item-level uncertainty and a clear separation between general awareness and reported household practices. Familiar ventilation and smoke concepts were recognized more readily than less visible mechanisms such as formaldehyde, residual tobacco-smoke exposure and sick-building-syndrome concepts. Mitigation behaviors coexisted with frequent source-related practices, particularly gas-stove use, air-freshener use, pesticide use and indoor smoking. Preventive attitudes were more consistently associated with lower source-frequency and lower odds of regular indoor smoking than knowledge alone, although the cross-sectional design precludes causal interpretation. The findings provide an initial evidence base for developing and testing locally adapted, source-specific indoor-air communication rather than relying only on generic awareness messages. Representative studies with objective exposure measurements, validated instruments and longitudinal or intervention designs are needed before population prevalence or causal conclusions can be drawn.

## Data Availability

The raw data supporting the conclusions of this article will be made available by the authors, without undue reservation.
